# Some Architectural and Sanitation Pamphlets

**Published:** 1913-02-15

**Authors:** 


### SOME ARCHITECTURAL AND SANITATION PAMPHLETS.

The year 1912 was not prolific in the output of books closely relating to hospital architecture and construction, using the words in their widest meaning, but the actual output testified to a sincere desire in all branches to make architecture the handmaid of treatment in each of the various departments of institutional work. Many papers have been written about sanatoria and the treatment of tuberculosis, some good and some indifferent; but we can recommend for re-reading a paper and discussion on the provision of sanatorium benefit under the National Insurance Act, 1911. The paper was read at the Sanitary Association of Scotland Montrose Congress last autumn by Dr. Charles Templeman, M.D., D.Sc., Medical Officer of Health, Dundee, and has been preserved in the Transactions published by Hodge and Co. In the discussion Dr. Chalmers, Medical Officer of Health, Glasgow, gave some interesting information as to the cost of sanatoria. For instance, he is reported to have said: "Another question concerns the cost of sanatorium beds. It is stated in the Departmental Report, that these can be provided for £150. Let me give you some figures which are derived from one of the most recently opened sanatoria in this country (Scotland), a sanatorium built by public contribution and with the land given free. In it there are only sixty-five beds, and the cost of the actual buildings, wards and administrative buildings, together amounts to £153 per bed, while the total cost per bed for the complete sanatorium was £260 per bed. In this case every effort was made to eliminate superfluous expenditure, to have no undue elaboration, and to have good work done at reasonable cost. If sanatoria are to be built at £150 per bed, then our architects have not yet disclosed to us how this can be done."

Dr. Templeman also questioned if it would be found possible in Scotland to erect and equip sanatoria at the modest sum per bed mentioned in the Departmental Committee's report. These facts are particularly interesting, for it is generally admitted that building as well as the general commodities of life are less expensive in Scotland than in England.

Among other interesting papers read at the Montrose Congress and reported in the Transactions were: "The Position of Local Authorities under the Operation of Section 63 of the National Insurance Act, 1911," by Lennox Sellar, M.A., and "Are the Current Methods of Disinfection consistent with the Latest Knowledge of Infective Processes?" by Dr. Buchanan, County Medical Officer, Haddington.

### Intercepting Traps.

In the discussion following Dr. Buchanan's paper Mr. Peter Fyfe, Glasgow, reminded those present that it was stated about 1870, when the late King had typhoid fever at Buckingham Palace, that it was owing to a faulty drainage system, and since that day intercepting traps to disconnect by water seal the sewer gas from the drain system had been the order of the day. He then referred at some length to the Report of the Departmental Committee (Cd. 6359) on Intercepting Traps. This report was published last year, and caused much discussion at the Royal Sanitary Institute among experts. It was reviewed in our columns of November 23, 1912, p. 223. It will be remembered that this somewhat bulky report practically condemned the intercepting trap as a useful member of the drainage system.

Another interesting Government publication was the Report on Isolation Hospitals, by H. Franklin Parsons, M.D. This report particularly applied to the cost of construction of isolation hospitals, but incidentally contains much useful data on the subject. It is interesting to note that particular mention is made of temporary buildings, such as iron hospitals. Though apparently much cheaper than permanent buildings of brick or stone, the cheapness is apparent rather than real.

Some interesting papers on "Modern German Hospitals" appeared in the *Journal of the Royal Institute of British Architects* from the pen of Mr. William Milburn, and are to be obtained as reprints. They form a comprehensive survey of the subject, carried out in an able manner, and are well worthy of study. The publication of the sixteenth edition of "Practical Sanitation," by Mr. George Reid, M.D., D.P.H. (Griffin and Co.), in 1912, testifies to the usefulness of this volume. It includes chapters on water supply and pollutions, ventilating and warming, sewerage, sanitary fittings, refuse disposal, infection and disinfection, and food, and is a useful handbook most particularly to the student engaged in sanitary work.

